# Selected microRNA Expression and Protein Regulator Secretion by Adipose Tissue-Derived Mesenchymal Stem Cells and Metabolic Syndrome

**DOI:** 10.3390/ijms25126644

**Published:** 2024-06-17

**Authors:** Grzegorz Wystrychowski, Klaudia Simka-Lampa, Agnieszka Witkowska, Ewelina Sobecko, Aleksandra Skubis-Sikora, Bartosz Sikora, Ewa Wojtyna, Agnieszka Golda, Katarzyna Gwizdek, Marta Wróbel, Łukasz Sędek, Sylwia Górczyńska-Kosiorz, Nikola Szweda-Gandor, Wanda Trautsolt, Tomasz Francuz, Celina Kruszniewska-Rajs, Joanna Gola

**Affiliations:** 1 Department of Nephrology, 4th Provincial Hospital, 41-902 Bytom, Poland; witkowskaaga@op.pl; 2 Department of Biochemistry, Faculty of Medical Sciences in Katowice, Medical University of Silesia, 40-055 Katowice, Poland; ksimka@sum.edu.pl (K.S.-L.); esobecko@sum.edu.pl (E.S.); tfrancuz@sum.edu.pl (T.F.); 3 Department of Histology and Embryology, Faculty of Medical Sciences in Katowice, Medical University of Silesia, 40-055 Katowice, Poland; askubis@sum.edu.pl (A.S.-S.); bsikora@sum.edu.pl (B.S.); 4 Institute of Medical Sciences, University of Opole, 45-040 Opole, Poland; ewa.wojtyna@uni.opole.pl; 5 Alfamed General Practice, 41-100 Siemianowice Slaskie, Poland; agziolk@gmail.com; 6 Department of Rehabilitation, Faculty of Health Sciences in Katowice, Medical University of Silesia, 40-055 Katowice, Poland; kgwizdek@sum.edu.pl; 7 Department of Internal Medicine, Diabetology and Cardiometabolic Diseases, Faculty of Medical Sciences in Zabrze, Medical University of Silesia, 40-055 Katowice, Poland; mwrobel@sum.edu.pl; 8 Department of Microbiology and Immunology, Faculty of Medical Sciences in Zabrze, Medical University of Silesia, 40-055 Katowice, Poland; lsedek@sum.edu.pl; 9 Department of Internal Medicine, Diabetology and Nephrology, Faculty of Medical Sciences in Zabrze, Medical University of Silesia, 40-055 Katowice, Poland; skosiorz@sum.edu.pl (S.G.-K.); nszweda@sum.edu.pl (N.S.-G.); wtrautsolt@sum.edu.pl (W.T.); 10 Department of Molecular Biology, Faculty of Pharmaceutical Sciences in Sosnowiec, Medical University of Silesia, 40-055 Katowice, Poland; ckruszniewska@sum.edu.pl (C.K.-R.); jgola@sum.edu.pl (J.G.)

**Keywords:** stem cells, mesenchymal stem cells, mesenchymal stromal cells, adipose tissue, obesity, metabolic syndrome, obesity, microRNA, cholesterol, IL-10, miR-155, IGF-1

## Abstract

The role of adipose mesenchymal stem cells (Ad-MSCs) in metabolic syndrome remains unclear. We aimed to assess the expression of selected microRNAs in Ad-MSCs of non-diabetic adults in relation to Ad-MSC secretion of protein regulators and basic metabolic parameters. Ten obese, eight overweight, and five normal weight subjects were enrolled: 19 females and 4 males; aged 43.0 ± 8.9 years. Ad-MSCs were harvested from abdominal subcutaneous fat. Ad-MSC cellular expressions of four microRNAs (2^−ΔCt^ values) and concentrations of IL-6, IL-10, VEGF, and IGF-1 in the Ad-MSC-conditioned medium were assessed. The expressions of miR-21, miR-122, or miR-192 did not correlate with clinical parameters (age, sex, BMI, visceral fat, HOMA-IR, fasting glycemia, HbA1c, serum lipids, CRP, and eGFR). Conversely, the expression of miR-155 was lowest in obese subjects (3.69 ± 2.67 × 10^−3^ vs. 7.07 ± 4.42 × 10^−3^ in overweight and 10.25 ± 7.05 × 10^−3^ in normal weight ones, *p* = 0.04). The expression of miR-155 correlated inversely with BMI (sex-adjusted r = −0.64; *p* < 0.01), visceral adiposity (r = −0.49; *p* = 0.03), and serum CRP (r = −0.63; *p* < 0.01), whereas it correlated positively with serum HDL cholesterol (r = 0.51; *p* = 0.02). Moreover, miR-155 synthesis was associated marginally negatively with Ad-MSC secretion of IGF-1 (r = −0.42; *p* = 0.05), and positively with that of IL-10 (r = 0.40; *p* = 0.06). Ad-MSC expression of miR-155 appears blunted in visceral obesity, which correlates with Ad-MSC IGF-1 hypersecretion and IL-10 hyposecretion, systemic microinflammation, and HDL dyslipidemia. Ad-MSC studies in metabolic syndrome should focus on miR-155.

## 1. Introduction

Adipose tissue is a rich source of mesenchymal stromal/stem cells (Ad-MSCs), which exhibit multipotent differentiation potential. In vitro, they are capable of differentiating into cells of not only the mesodermal but also the endodermal and ectodermal lineages. The direction of differentiation is dependent on environmental conditions. Mesenchymal stem cells do not express HLA class II antigens [1], which renders them suitable for allogeneic transplantations. When exogenous mesenchymal stem cells are administered in large quantities, they exhibit tropism to inflamed or necrotic tissues, exerting anti-apoptotic, oxidative stress-reducing, anti-fibrotic, and anti-inflammatory effects [2,3]. This is achieved through the secretion of growth factors, anti-apoptotic factors, cytokines, and microRNAs, either in the free state or within extracellular vesicles. Furthermore, the secretome of Ad-MSCs has been demonstrated to exert immunosuppressive effects. These effects include increases in the pool and activity of regulatory T cells; inhibition of NK cells, Th17, and B lymphocytes; activation of tolerogenic dendritic cells; and induction of Th1→Th2 and M1→M2 conversions [4].

In situ, Ad-MSCs are unipotent and constitute a potential source of preadipocytes. However, their role as local regulators is being increasingly discovered [5]. It has been found that Ad-MSCs display two opposite phenotypes with regard to cell-to-cell and secretory effects: a pro- or anti-inflammatory one, depending on the stimulating factors [6]. In the course of obesity and type 2 diabetes, Ad-MSCs exhibit pro-inflammatory characteristics, with increased expression of NLRP3 inflammasome elements [7], secretion of monocyte chemoattractant protein-1, IL-6, and IL-8 [7,8], and activation of macrophages and Th17 lymphocytes [9], which collectively lead to microinflammation of adipose tissue. In addition, several studies have demonstrated a reduction in the proliferative and differentiation potential of Ad-MSCs or preadipocytes in diabetic subjects compared to non-diabetic individuals. This was associated with subsequent fat hypertrophy, which prevails in obesity over healthier hyperplasia [10,11]. Furthermore, recent studies have shown that the secretome of Ad-MSCs from obese subjects becomes enriched in adipokines such as leptin [12].

Among the Ad-MSC-secreted compounds that can be involved in the development or complications of metabolic syndrome is vascular endothelial growth factor (VEGF) [13]. It was revealed in animal models that induced fat tissue overexpression of VEGF increases local vasculature and promotes adipocyte beiging. Conversely, inhibition of the VEGF receptor in adipocytes aggravates insulin resistance in rats exposed to a high-fat diet [14,15]. Another growth factor of interest secreted by Ad-MSCs is insulin-like growth factor 1 (IGF-1) [13]. IGF-1 stimulates preadipocyte differentiation, and although mature adipocytes do not preserve IGF-1 receptor expression, they can secrete it [16]. IGF-1 exerts a protective effect against insulin resistance, likely through the inhibition of growth hormone secretion and its insulin-antagonizing effects in the liver and adipose tissue (higher gluconeogenesis and free fatty acid release into blood, respectively) [16]. Consequently, decreased circulating levels of IGF-1 are independently predictive of and present in metabolic syndrome [17,18].

Amongst cytokines, both the major pro-inflammatory interleukin 6 (IL-6) and anti-inflammatory interleukin-10 (IL-10) were reported to be secreted by Ad-MSCs [13]. Theoretically, IL-6 may propagate the adipose tissue microinflammation that typically occurs in obesity. Indeed, circulating IL-6 levels and IL-6 expression in white adipose tissue are increased in the course of obesity. A significant portion of the production site is adipocytes, yet the impact of IL-6 is variable. In adipose tissue, IL-6 stimulates pro-inflammatory signals in T cells, whereas if bound classically to IL-6 receptors in macrophages, it exerts mainly anti-inflammatory actions with M2 polarization [19,20]. Nevertheless, IL-6 can also directly participate in the pathogenesis of the metabolic syndrome by enhancing lipolysis in the visceral fat and thus contributing to liver steatosis [21]. The role of IL-10 in metabolic syndrome may be associated with anti-inflammatory effects on the one hand, and with direct modulation of key metabolic pathophysiology on the other. It was revealed that IL-10 inhibits the thermogenesis of adipocytes and subsequently hinders the beiging of white adipocytes in mice [22]. Furthermore, it was demonstrated in mice that the source of IL-10 with such an effect is regulatory T cells [23]. The more abundant Ad-MSCs have not yet been studied for this function.

MicroRNAs are 21–23 base-pair double-strand particles of non-coding RNA that bind to mRNAs and regulate their translation. They are relatively stable in the body fluids [24], and their role as local and distant regulators of homeostasis is being extensively studied. A growing number of studies have demonstrated changes in the concentrations of circulating microRNAs in the course of obesity or metabolic syndrome [24]. Among these microRNAs, miR-21 [25], miR-122 [26], miR-155 [27], and miR-192 [28] have been shown to be associated with adipose tissue inflammation, insulin resistance, or liver steatosis. miR-21 has been identified as a protective particle, while the others have been demonstrated to have largely detrimental effects. Although Ad-MSCs are known to secrete microRNAs, the role of these microRNAs in obesity remains unclear. Should such a contribution be revealed and the respective hypo- or hypersecreted microRNAs be identified, the administration of respective mimics or antagonists could be conceived as a therapeutic option in the future. It has been reported that relatively large amounts of miR-21 are present in mesenchymal stem cells and within their exosomes [29,30]. Furthermore, miR-122 has been found plentiful in the exosomes of bone marrow-derived mesenchymal stem cells in a recent Canadian study [31]. Cytoplasmic miR-155 was identified as a key microRNA inducer of the immunosuppressive effects of human bone marrow-derived mesenchymal stem cells [32], while exosomal miR-192 derived from rat Ad-MSCs demonstrated the ability to mitigate retinal inflammation and angiogenesis in rats with diabetic retinopathy [33].

In accordance with the aforementioned rationale, the objective of this study was to assess whether the Ad-MSC expression of the four microRNAs (miR-21, miR-122, miR-155, and miR-192) and secretion of the four protein regulators (VEGF, IGF-1, IL-6, and IL-10), with potential involvement in the pathogenesis of metabolic syndrome, are associated with body weight and basic metabolic parameters in a sample of non-diabetic adults. Additionally, this study aimed to determine whether these microRNA expressions and protein secretions are related to each other.

## 2. Results

The study group consisted of 19 female and 4 male adults, with a mean age of 43.0 ± 8.9 years (ranging from 28 to 63 years). There were 10 obese, 8 overweight, and 5 normal weight subjects. Six female obese subjects were prediabetic, according to fasting serum glucose concentration of 100–125 mg/dL or HbA1c fraction of 5.7–6.4%.

### 2.1. Basic Clinical Parameters

Explicably, the specified body mass index groups exhibited differences in metabolic syndrome features. The subjects with elevated BMI exhibited higher rates of high blood pressure, hyperglycemia, and low HDL cholesterolemia. Additionally, their visceral fat volume was greater, and their serum CRP concentration was higher (Table 1). Six obese prediabetic females did not differ statistically significantly from four obese euglycemic females with regard to any of the clinical parameters. The exception was a statistically significant difference in HbA1c fraction, with the obese prediabetic females exhibiting a mean value of 5.78 ± 0.17% and the obese euglycemic females exhibiting a mean value of 5.42 ± 0.25% (*p* = 0.04).

### 2.2. Cytometric Ad-MSC Verification

**Figure 1:**
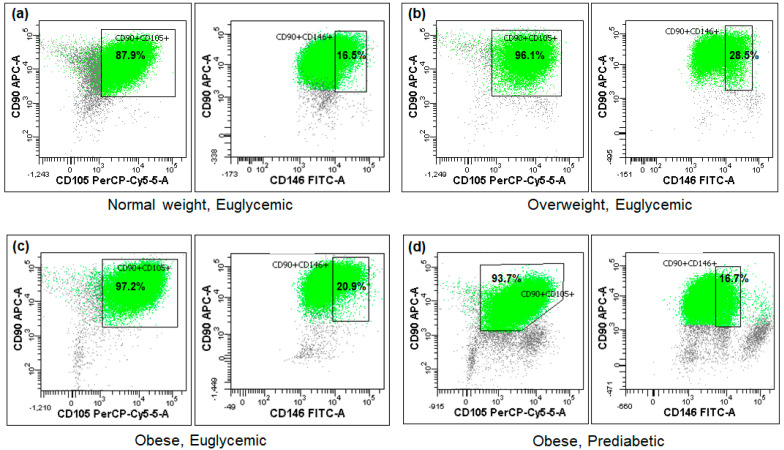
Fractions of the analyzed cells positive for CD90, CD105, and CD146 mesenchymal stem cell surface markers in four exemplary subjects (**a**–**d**) differing in BMI or glycemic status (flow cytometry).

The cytometric analysis demonstrated that the tested Ad-MSC samples exhibited minimal expression of the CD45 antigen, whereas the mean expression of CD90 approached 98% and that of CD105 reached 96%. Conversely, the expression of the CD146 surface marker was observed in less than 1/5 of the sample cells on average (Figure 1, Table 2). No statistically significant differences were observed between the BMI or glucose metabolism subgroups (Table 2), nor were any associations found with the studied miRNA expressions or other parameters.

### 2.3. Expression of microRNAs

Among the four microRNAs studied, miR-21 exhibited the most abundant expression in Ad-MSCs. The expression of miR-155 was approximately 1000 times lower, while those of miR-122 and miR-192 were approximately 10,000 lower.

#### 2.3.1. Expression of microRNAs across the Studied Subgroups

**Figure 2:**
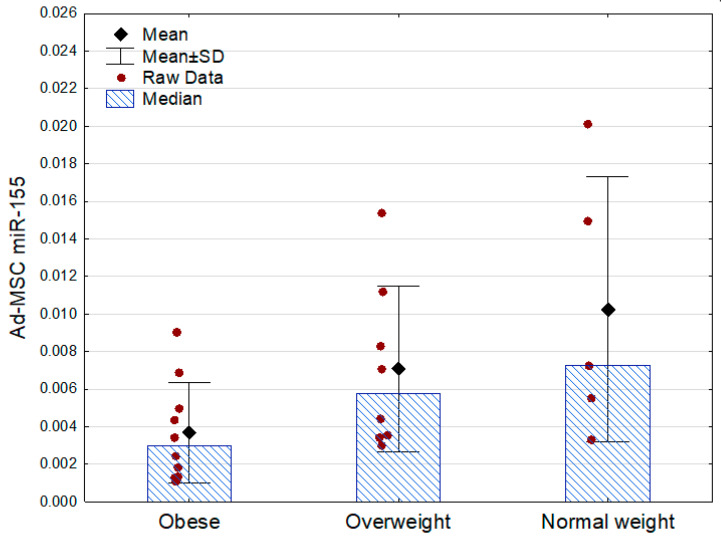
Ad-MSC expression of miR-155 (2^−ΔCt^ values related to SNORD61 control) in the obese, overweight, and normal weight subjects (*p* = 0.04 between the groups, Kruskal–Wallis ANOVA; Mann–Whitney U test as post hoc, overweight vs. normal weight: *p* = 0.51). SD—standard deviation.

Only miR-155 demonstrated associations with the other analyzed parameters. Its expression was lowest in the obese subjects, being approximately two times lower than in the subjects who were overweight and three times lower than in the normal weight participants. There were no statistically significant differences between the obese prediabetic and euglycemic subjects (Table 3, Figure 2).

#### 2.3.2. Expression of microRNAs in Relation to Clinical Parameters

**Figure 3:**
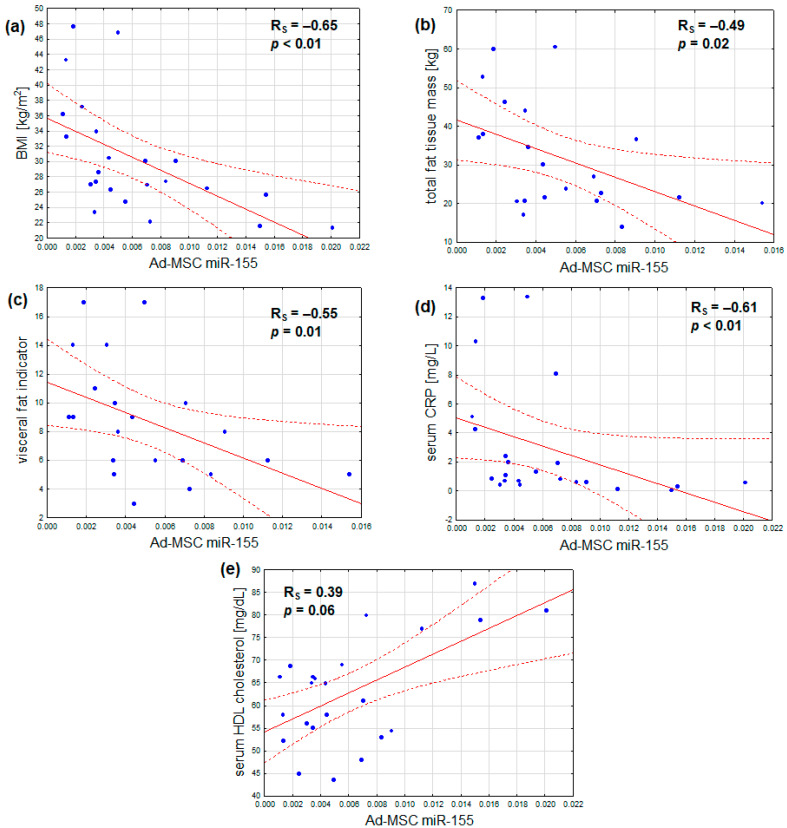
Inverse correlations of Ad-MSC expression of miR-155 and (**a**) body mass index, (**b**) total fat tissue mass, (**c**) visceral fat indicator, and (**d**) serum CRP concentration; (**e**) a direct correlation of Ad-MSC expression of miR-155 and serum HDL cholesterol concentration (scatterplots with regression lines and their 95% confidence intervals (dotted lines); statistical characteristics according to Spearman rank correlation analysis).

In addition, the expression of miR-155 was found to correlate not only with BMI, but also inversely with total fat mass, visceral adiposity, and serum CRP. Additionally, a marginal positive correlation was observed between serum HDL cholesterol and miR-155 expression. No notable correlations were identified between miR-155 expression and age, serum fasting glucose, HbA1c, insulin resistance, serum LDL cholesterol, and triglyceride concentrations, or estimated glomerular filtration (Table 4, Figure 3a–e). After adjustment for sex, the outcomes remained similar, with the exception that the association between Ad-MSC miR-155 expression and serum HDL cholesterol became statistically significant (Table 4).

### 2.4. Secretion of Protein Regulators

With regard to the analyzed secretions of Ad-MSCs, the most abundant in terms of the number of particles was that of IGF-1 (209.2 ± 62.9 fmol/L, ranging from 117.6 to 317.6), followed by IL-6 (87.3 ± 30.6 fmol/L, ranging from 14.3 to 166.7) and VEGF (75.4 ± 23.4 fmol/L, ranging from 32.5 to 104.8). IL-10 exhibited a markedly lower output (0.04 ± 0.02 fmol/L, ranging from 0.02 to 0.09).

#### 2.4.1. Secretion of Protein Regulators across the Studied Subgroups

The secretion of the studied growth factors and interleukins by Ad-MSCs did not differ statistically significantly between the designated groups. However, there was a small trend of higher IGF-1 secretion in the obese subjects than in the normal weight ones (Table 5).

#### 2.4.2. Secretion of Protein Regulators in Relation to Clinical Parameters

The results of this study indicate several statistically significant univariate correlations between the secretions of the studied protein regulators and metabolic parameters. The secretion of VEGF was inversely correlated with fasting glycemia, whereas that of IGF-1 was directly related to body weight, BMI, visceral adiposity, and serum CRP concentration. In contrast, the production of IL-6 increased with age and reduced estimated glomerular filtration. The secretion of IL-10 was not statistically significantly associated with any of the studied parameters (Table 6). After adjusting for sex, the associations revealed were further enriched with a negative relationship between Ad-MSC VEGF production and body fat tissue fraction, positive correlations between Ad-MSC secretion of IGF-1 and fasting glycemia or total fat tissue mass (marginally), as well as a negative association between IGF-1 release and serum HDL cholesterol at *p* = 0.08 (Table 6).

### 2.5. Expression of microRNAs vs. Secretion of Protein Regulators

**Figure 4:**
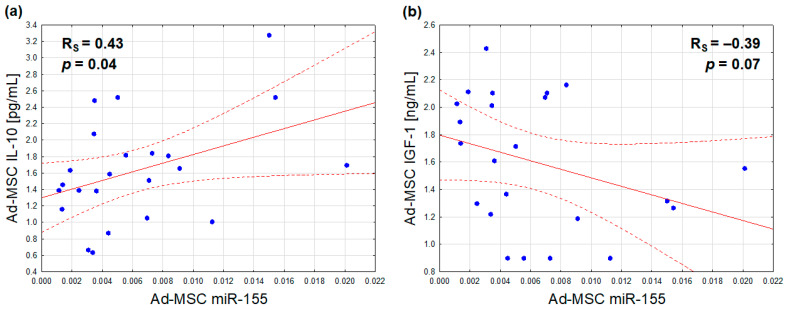
Correlations of the Ad-MSC expression of miR-155 (2^−ΔCt^ values related to SNORD61 control) with Ad-MSC secretions of IL-10 (**a**) and IGF-1 (**b**) (Ad-MSC-conditioned medium concentrations) (scatterplots with regression lines and their 95% confidence intervals (dotted lines); statistical characteristics according to Spearman rank correlation analysis).

With regard to the relationships between the analyzed Ad-MSC microRNA expressions and protein secretions, only miR-155 synthesis was found to be positively associated with the secretion of IL-10 (Figure 4a), and marginally, inversely, with that of IGF-1 (Figure 4b). After adjusting for sex, the former association exhibited a slight attenuation (r = 0.40, *p* = 0.06), while the latter exhibited a slight improvement (r = −0.42, *p* = 0.05)

## 3. Discussion

### 3.1. Ad-MSC Antigenic Characteristic

Cytometric analysis of the Ad-MSC phenotype demonstrated the expected negativity for the CD45 antigen and positivity for the CD90 and CD105 antigens. Additionally, only a minority of the cells expressed the CD146 surface protein. This is consistent with the findings of other studies, which have demonstrated that the proportion of mesenchymal stem cells expressing CD146 varies depending on their origin and is relatively small in adipose tissue [34,35,36]. In general, CD146-negative Ad-MSCs are located more distantly from blood vessels and have diminished proliferative and differentiation potentials [37,38]. There were no associations between the percentage of CD146-positive cells and the studied parameters, which suggests that this antigenic characteristic is not related to microRNA expression and metabolic disturbances in non-diabetic subjects.

### 3.2. Decreased Ad-MSC Expression of miR-155 as a Hallmark/Source of Obesity and Metabolic Microinflammation

The obtained results indicate that the Ad-MSC microRNA synthesis is altered in the course of obesity with regard to miR-155. The expression of miR-155 is decreased in parallel to visceral fat excess, elevated serum CRP, and decreased serum HDL cholesterol concentrations. Furthermore, the outcomes suggest that Ad-MSC hypoexpression of miR-155 is linked to decreased secretion of IL-10 and increased production of IGF-1 by these cells. The latter appears to be associated with metabolic disturbances.

#### 3.2.1. IL-10 as a Possible Anti-Inflammatory Effector of Ad-MSC miR-155

The authors of numerous works have demonstrated that miR-155 is a pro-inflammatory regulator of cytokine expression in inflammatory cells [39,40]. Its synthesis is responsive to various inflammatory stimuli. It increases the expression of pro-inflammatory interleukins, M1 polarization of macrophages, and Th17 commitment of CD4+ T cells [41,42,43]. In line with these observations, the main anti-inflammatory cytokine, IL-10, has been shown to suppress LPS-induced expression of miR-155 in inflammatory cells. B lymphocytes [44] and macrophages [45] are two cell types in which IL-10 inhibits the maturation of miR-155 transcripts.

Our findings indicate a positive association between Ad-MSC IL-10 secretion and miR-155 expression, which is contrary to previous observations. This suggests that there may be an opposite relationship in these cells, whereby miR-155 enhances IL-10 synthesis in an autocrine manner. Such an interaction has been demonstrated in regulatory IL-10-producing B lymphocytes, in which miR-155 mimic increased, whilst miR-155 inhibitor reduced, IL-10 expression by respectively relieving or inducing histone methylation repression of IL-10 transcription [46]. It may be assumed that oversecretion of IL-10 by Ad-MSCs would be beneficial by attenuation of the adipose tissue inflammation. As previously stated, the role of IL-10 in adipose tissue remains unclear. Early studies have demonstrated increased fat expression of IL-10 following weight loss with bariatric surgery [47] or low-calorie diets in humans [48]. Subsequent studies demonstrated elevated IL-10 expression in adipose tissue macrophages in obese subjects. However, in contrast to mice, IL-10 did not influence thermogenesis in human adipocytes. It was postulated that overexpression of IL-10 was a counter-regulatory response to inflammation of the adipose tissue [49]. Nevertheless, a substantial paracrine function of Ad-MSC IL-10 is negated by its limited secretion from the stromal vascular fraction in comparison to that from adipose tissue macrophages, as demonstrated in the aforementioned study [49]. Furthermore, no correlations were observed between Ad-MSC IL-10 secretion and the clinical parameters in our study, and a markedly lower production compared to the largely pro-inflammatory IL-6 also casts doubt on the clinical significance of this source of IL-10.

#### 3.2.2. Ad-MSC miR-155 as a Primer of Adipose Tissue Regulatory T Cells

Alternatively, Ad-MSC miR-155 may prevent obesity in a more direct way. Notably, miR-155 has been found to be crucial for the development of regulatory T cells (Tregs). Two independent studies on mice from 2009 demonstrated that the transcription factor Foxp3 activates the expression of miR-155 in Tregs, which is essential for the proliferation of these cells and the maintenance of their physiological subset size [50,51]. A recent study by a Polish group from Gdańsk demonstrated that human Ad-MSCs are capable of priming the Tregs’ inhibition of other T cells’ proliferation. This was achieved in two ways: either by co-culturing the Ad-MSCs and Tregs or by exposing the Tregs to the Ad-MSC supernatants. In the former case, a transfer of mitochondria was detected between the cells, which correlated with a longer Foxp3 expression preservation in Tregs in vitro [52].

As is known from other studies, lower quantities of Tregs within the adipose tissue are observed in obese subjects and correlate with insulin resistance [53]. Consequently, our findings indicate that the reduced expression of miR-155 in Ad-MSCs and the subsequent diminished transfer of this microRNA from Ad-MSCs to neighboring Tregs may disrupt the function and/or number of Tregs. This, in turn, may lead to the development of obesity and metabolic syndrome. To substantiate this hypothesis, further studies are required to assess the transfer of miR-155 between Ad-MSCs and Tregs derived from obese versus lean subjects.

#### 3.2.3. Ad-MSC miR-155 as an Autocrine Inhibitor of Adipogenesis

It has been demonstrated that one of the major pro-inflammatory cytokines, TNFα, increases miR-155 expression in white preadipocytes and adipocytes through the NFκB pathway [54,55]. It is noteworthy that this overexpression of miR-155 was found to inhibit the expression of several genes involved in adipogenesis and lipogenesis in fat cells, including fatty acid synthase, acetyl coenzyme A carboxylase, sterol responsive element binding protein 1c, and stearoyl coenzyme A desaturase. Consequently, preadipocyte differentiation was inhibited [54,55], and adipocytes exhibited features of dedifferentiation, with reduced volumes of lipid droplets [55]. This is corroborated by the findings of another group, which revealed that miR-155 expression in subcutaneous adipose tissue correlates inversely with mean adipocyte volume [56]. With reference to our findings, it may be deduced that miR-155 overexpression in Ad-MSCs inhibits their differentiation into fat lineage cells and thus prevents adipose tissue expansion and fat accumulation in response to inflammatory stimuli.

In addition to the aforementioned findings, Karkeni et al. observed an elevated overall miR-155 expression in adipose tissue from obese subjects compared to lean ones [55]. However, this does not necessarily contradict our results, as an increased synthesis of miR-155 may occur in macrophages infiltrating adipose tissue during the course of obesity, as previously discussed. Furthermore, other groups have obtained contradictory results pertaining to obesity complicated by metabolic syndrome. In obese subjects with type 2 diabetes, the subcutaneous fat expression of miR-155 was found to be lower than in normoglycemic obese counterparts [56]. In another study, circulating levels of miR-155 were found to be lower in obese subjects with metabolic syndrome, compared to healthy obese subjects, and correlated negatively with circulating adipogenic transcription factor mRNA [57].

#### 3.2.4. Ad-MSC miR-155 as an Inhibitor of IGF-1

Another potential mechanism by which Ad-MSC miR-155 may protect against visceral obesity is through the autocrine inhibition of IGF-1 release with subsequent prevention of its detrimental effects on the metabolism. It is likely that this action of Ad-MSC-derived IGF-1 is local, with no relation to the opposite findings regarding circulating IGF-1 [16,17,18]. IGF-1 is known to participate in adipogenesis; in rats, it was demonstrated to enhance the differentiation of mesenteric stromal vascular cells into mesenteric visceral adipocytes in a synergistic manner with insulin [58]. Scavo et al. demonstrated that IGF-1 enhances the proliferation and maturation of human mesenchymal stem cells into adipocytes, as well as lipid accumulation throughout the process [59]. The correlations of Ad-MSC-secreted IGF-1 with visceral obesity, higher fasting glycemia, systemic microinflammation, and, to a lesser extent, lower serum HDL cholesterol in our subjects appear to support the obesogenic influence of this cytokine in adipose tissue and the benefits of reducing its secretion by miR-155. A mechanism of downregulating IGF-1 expression by miR-155 was recently demonstrated by Shen et al. in murine colonic myocytes, where it was shown that miR-155 binds and suppresses the translation of IGF-1 mRNA [60].

### 3.3. Significance of Ad-MSC IL-6 and VEGF

The results of this study indicate that Ad-MSC secretion of the major pro-inflammatory interleukin (IL-6) increases with older age and the onset of kidney function impairment. However, the interpretation of this finding is challenging due to the interdependence of the two latter variables. Nevertheless, an additional adjustment for age did not eliminate the partial correlation between Ad-MSC secretion of IL-6 and eGFR (r = −0.41, *p* = 0.03). This suggests that kidney disease does affect the secretome of Ad-MSCs. In support of this, it is known that uremic toxins, such as intestinal bacterial products, may induce inflammation of adipose tissue. Indoxyl sulfate has been demonstrated to increase oxidative stress and the expression of monocyte chemoattractant protein-1 in murine adipocytes [61]. Furthermore, p-cresol and indoxyl sulfate have been shown to alter the protein secretome and impair the osteogenic differentiation potential of human bone marrow-derived mesenchymal stem cells [62,63]. It is questioned if Ad-MSC IL-6 hypersecretion due to renal dysfunction would contribute solely to adipose tissue inflammation or to malnutrition–inflammation–atherosclerosis syndrome.

The outcomes of this study also indicate a reduction in VEGF release from Ad-MSCs at the onset of glycemic disturbances. In light of the weaker associations between Ad-MSC VEGF secretion and obesity markers compared to that with fasting glycemia, it can be inferred that the slightly hyperglycemic milieu impairs VEGF secretion, rather than the reverse. Other studies have reported decreased Ad-MSC secretion of VEGF in diabetic patients [64], which may contribute to diminished vascularization of adipose tissue [65]. The results of this study suggest that this process may already be initiated at the prediabetic stage.

### 3.4. Study Limitations

It is important to note that the cross-sectional nature of our study precludes a firm judgment on the causality or direction of the relationships between the Ad-MSC miR-155 and the obesity-related parameters. It cannot be excluded that the observed hypoexpression of miR-155 in Ad-MSCs in obese, inflamed, or dyslipidemic milieus is not a contributor to these metabolic disturbances, but rather an aftermath of them. Alternatively, it may be that miR-155 hypoexpression is a counteraction that develops in response to these disturbances. It may also be that both of these scenarios are true and coexist.

In a study by Alicka et al. [64], the secretome of Ad-MSCs derived from six obese type 2 diabetic patients was found to differ from that of six normal weight non-diabetic controls. Along with reduced viability and proliferative potential, Ad-MSCs from obese diabetic individuals differed in the secretion of six studied microRNAs (different from ours), secreted less VEGF and CXCL12 (stromal cell-derived factor-1), but more leptin. Nevertheless, the study was limited in size and, as a cross-sectional study, was unable to conclude causality. Furthermore, it appears that the diabetic environment, with its accompanying oxidative stress, is the primary factor affecting Ad-MSCs. This is consistent with our intention of not including diabetic subjects, as the majority of studies have demonstrated a less favorable phenotype of stem cells cultured in a high-glucose environment compared to control cells [66]. In the context of high-glucose conditions, mesenchymal stem cells demonstrated reduced potency for proliferation and differentiation (with the exception of adipogenicity) [67], inferior regenerative effects, and altered growth factor and microRNA secretomes [68,69]. Finally, studies employing the most optimal approach to examine the direction and causality of the relationships between metabolic disturbances and Ad-MSC secretome are still lacking. These would involve longitudinal assessment of Ad-MSC secretions in experimental animals prior to and after the induction of pre- and overt diabetes or hyperlipidemia.

It is also important to acknowledge that our methodology for assessing microRNAs in Ad-MSCs, and not in their conditioned media or extracellular vesicles, means that our results cannot be fully translated to studies of the microRNA secretome. However, our approach allows the inclusion of microRNAs that may be transferred to neighboring cells within cytoplasmic fragments or organelles, as demonstrated by Piekarska et al. in the aforementioned study of Ad-MSC and Treg interactions [52].

It is regrettable that the isolation of Ad-MSCs was not optimal, as it relied on the specific culture conditions and did not include an immunoselection of the cells. This was due to the unavailability of relevant sorting machinery. Nevertheless, despite this drawback, the cytometric verification of the Ad-MSC phenotype proved that our cell cultures consisted almost solely of Ad-MSCs, as presented and discussed above.

A notable limitation of this study is the relatively small number of subjects studied (although within the range of most stem cell research). While the intention was to include at least 50 subjects, this was impeded by difficulties with subject recruitment and logistical problems due to the outbreak of the COVID-19 pandemic. Nevertheless, the outcomes obtained in a sample of 23 cases are deemed sufficient to encourage further studies on the involvement of Ad-MSC microRNAs in metabolic syndrome, with a particular focus on the role of miR-155. In future studies, it would be of significant interest to ascertain whether the microRNA and protein secretome profiles of Ad-MSCs translate into the respective circulating blood levels. A larger sample size will enable the differentiation of the effects of obesity from those of its complications, as well as the elimination of potential biases associated with pharmacological treatments.

Furthermore, it is important to acknowledge the skewed sex distribution of our study group. This was due to a much lesser interest in study participation from the approached men. However, four male subjects, all of whom were overweight, did not differ statistically significantly from the four female overweight counterparts in any of the studied parameters. The sole exception was a higher fraction of fat tissue in relation to body weight in the female subjects (33.8% vs. 23.6%, *p* = 0.03). Notably, the two groups were not statistically significantly heterogeneous with regard to visceral adiposity (6.0 ± 1.4 vs. 8.0 ± 4.9, respectively, *p* = 0.88), nor the analyzed Ad-MSC microRNAs and secreted proteins (*p* values ranging from 0.3 to 0.9). Furthermore, to mitigate the risk of a statistical bias resulting from the female predominance of the study group, an adjustment for sex was made in the studied relationships using a partial correlation test, as outlined below. The outcomes were not substantially different from those of the univariate Spearman correlation analyses, with a notable exception of those involving serum HDL cholesterol and Ad-MSC secretion of IGF-1. The former is understandable in view of the generally higher serum HDL cholesterol concentration in women, which is reflected in the different diagnostic criteria of HDL dyslipidemia depending on sex [70]. The latter observation may be linked to the reported slight differences in the circulating levels of IGF-1 between the sexes [71].

## 4. Materials and Methods

### 4.1. Study Participants and Assessment of Basic Clinical Parameters

Adult non-diabetic subjects were enrolled in this study. The participants were recruited from among patients of general practices and healthy volunteers. To be eligible for this study, participants were required to meet the following criteria: (1) they could not be affected with an overt inflammatory, cancerous, or endocrine disease; (2) they could not have stage G3-G5 chronic kidney disease; and (3) they could not have been treated with glucose- or lipid-lowering drugs.

All subjects underwent measurement of their basic glucose and lipid metabolism parameters, as well as serum creatinine and C-reactive protein (CRP) concentrations, in fasting venous blood samples at a commercial laboratory. The glomerular filtration rate was estimated with the CKD-EPI formula. The total fat mass percentage and visceral fat indicator, as an estimate of visceral fat volume, were assessed using a bioimpedance device (Tanita BC-418, Tanita Corporation, Tokyo, Japan).

### 4.2. Ad-MSC Culture

Within one week of the blood collection, the study subjects underwent the suction of a 5–10 mL sample of abdominal subcutaneous adipose tissue for the sole purpose of this research. The adipose tissue samples were rinsed several times with an antibiotic solution and then fragmented using a scalpel. The shredded tissue was placed in a Falcon tube containing 0.1% collagenase type I in Dulbecco’s Phosphate-Buffered Saline (dPBS) solution containing 1% bovine serum albumin. Fat tissue digestion was carried out in a shaker at 200 RPM at 37 °C for 1–2 h until a homogeneous suspension was obtained. Subsequently, collagenase activity was inhibited by the addition of a 10% fetal bovine serum solution in phosphate-buffered saline. The solution was then centrifuged at 1200 RPM for 5 min at room temperature to separate the phases, and the upper and middle layers were discarded. The remaining stromal vascular cell pellet, which comprises Ad-MSCs, preadipocytes, erythrocytes, and undigested collagen fibers, was suspended in Dulbecco’s modified Eagle’s medium supplemented with 10% fetal bovine serum and 1% antibiotic solution (penicillin, streptomycin, and amphotericin B), and transferred to a culture dish. The culture was performed under standard conditions: the temperature was maintained at 37 °C, the humidity was 95%, and the atmosphere was saturated with 5% CO_2_.

The cells that adhered to the culture dish constituted the Ad-MSC population. The medium was changed, and microscopic observations were carried out every two days. Prior to changing the culture medium, the dish was rinsed several times with dPBS solution to remove non-adherent cells (mainly erythrocytes) and remnants of undigested tissue, such as collagen fibers. Cells from the first passage were stored in liquid nitrogen for further research. Following the collection of all samples, the cells were thawed and the cell culture was continued under standard conditions, as described above. The cell culture of the fourth passage was used for the experiment. The medium was renewed for the last time 72 h prior to the isolation of RNA from the cell pellet and collection of conditioned medium for the study of protein secretions.

### 4.3. Assessment of microRNA Expression in Ad-MSCs

The extraction of total cellular RNA, including microRNAs, was performed using the miRNeasy Mini Kit (Qiagen, Valencia, CA, USA) according to the manufacturer’s protocol. The expressions of the 5p strands of miR-21, miR-122, miR-155, and miR-192 were assessed with reverse transcription quantitative polymerase chain reaction (RT-qPCR), with the use of the SNORD61 as a reference control. The cDNA was synthesized using the miScript II RT Kit (Qiagen, Valencia, CA, USA). The miScript SYBR Green PCR Kit and miScript Primer Assays were employed for the qPCR reaction: Hs_miR-21_2; Hs_miR-122a_1; Hs_miR-155_2; Hs_miR-192_1; and Hs-SNORD61_11 (Qiagen, Valencia, CA, USA). The reaction was conducted using the Light Cycler 480 System (Roche Life Science, Basel, Switzerland). Each test in this study was performed in duplicate. The expression level was defined based on the threshold cycle, and the relative expression of genes was analyzed by the 2^−ΔCt^ relative quantification method after normalization with the reference control.

### 4.4. Assessment of Ad-MSC Secretion of Protein Regulators

The Ad-MSC-conditioned medium was collected using sterile 0.22 µm syringe filters (Millex GV 13 mm, Merck Life Science, Darmstadt, Germany), centrifuged, and used for the assessment of the studied protein regulators. The concentrations of VEGF and IGF-1 (R&D Systems Inc., Minneapolis, MN, USA), IL-10 (Diaclone SAS, Besancon, France), as well as IL-6 (ThermoFisher Scientific, Waltham, MA, USA) were quantified in the media samples using dedicated ELISA kits, in accordance with the manufacturers’ instructions.

### 4.5. Cytometric Ad-MSC Verification

The mesenchymal stem cell phenotype was verified by examining the specific surface marker pattern in the last passage cell samples using a dedicated flow cytometry reagent (Human Mesenchymal Stem Cell Multi-Color Flow Kit, R&D Systems, Minneapolis, MN, USA). The kit contained four antibodies in total, including the three positive ones for Ad-MSCs, CD90, CD105, and CD146, and a negative one, CD45, as well as appropriate isotype controls for the antibodies used. These markers have been found to be discerning of mesenchymal stem cells, in addition to plastic adherence of the cells. However, the positivity of the cells regarding CD146 is not ubiquitous, as previously discussed [34,35,36]. Cells for cytometric analysis were prepared according to the manufacturer’s protocol and analyzed using the FACSCanto II flow cytometer (Becton Dickinson Biosciences, Franklin Lakes, NJ, USA).

### 4.6. Statistical Analysis

Statistical analyses were conducted with Statistica 14.0, TIBCO Software Inc. The χ^2^ test was applied to compare the distributions of sex and hypertension between the studied subgroups. Due to relatively small data samples, Kruskal–Wallis Analysis of Variance with the Mann–Whitney U test as the post hoc test was employed for the comparisons of the three groups differing in BMI, and the Mann–Whitney U test was used for the comparisons of the variables between the prediabetic and euglycemic subjects. The relationships between the continuous variables were assessed with the Spearman rank correlation analysis. In the context of a limited number of cases, this test appeared to be suitable, as it did not require the data to be normally distributed or linearly related. The value of the obtained Spearman correlation coefficient reflected the strength of the dependence between the variables under analysis, and its sign indicated the direction of the association. Furthermore, the relationships between the variables were assessed with adjustment for the possible confounding effect of sex, given the imbalance in the sex ratio of the study group. This was accomplished through the utilization of the partial correlation test with the prior transformation of the non-normally distributed variables to meet the requisite of normal distribution (*p* > 0.1 in the Lilliefors test). The natural logarithm transformation was applied to age, HOMA-IR, serum triglyceride and CRP concentrations, eGFR, and Ad-MSC expression of miR-21, miR-122, and miR-155. Conversely, square root transformation was employed for the Ad-MSC secretions of IGF-1 and IL-6. For all statistical analyses, a significance level of *p* < 0.05 was considered to indicate statistical significance, while a significance level of *p* = 0.05–0.07 was considered to indicate a marginal statistical significance.

## 5. Conclusions

In conclusion, the expression of miR-155 in adipose tissue-derived mesenchymal stem cells appears to be blunted in the context of visceral obesity. This hypoexpression of miR-155 is correlated with Ad-MSC IGF-1 hypersecretion and IL-10 hyposecretion, systemic microinflammation, and serum HDL hypocholesterolemia. Independently of the Ad-MSC expression of microRNAs under investigation, a reduction in the production of VEGF by Ad-MSCs appears to be linked to the development of prediabetes, while an increase in the secretion of IL-6 has been associated with the early stages of renal function impairment.

These findings highlight the need for further studies of the Ad-MSC secretome in the pathogenesis of metabolic syndrome, with a particular focus on miR-155, major growth factors, and interleukins. It would be beneficial to include research on the influence of the recently introduced hypoglycemic medication with body weight-reducing effects. Experimental studies using a miR-155 mimic should verify its causative role in the prevention of obesity and its complications.

Given their biological stability, microRNA mimics or antagonists may become a therapeutic option in the treatment of obesity and metabolic syndrome in the future.

## Figures and Tables

**Table 1:** Basic clinical parameters in the subjects divided into subgroups as per BMI (obese: ≥30 kg/m^2^; overweight: 25–29.9 kg/m^2^; normal weight: <25 kg/m^2^).

	All Subjects *n* = 23	Obese *n* = 10	Overweight *n* = 8	Normal Weight *n* = 5
Age [years]	43.0 ± 8.9 (28–63)	44.0 ± 9.0 (31–60)	41.3 ± 10.7 (28–63)	43.8 ± 6.6 (39–54)
Sex [M/F] *	4/19	0/10	4/4	0/5
Arterial hypertension [%] *	5 (21.7%)	4 (40.0%)	1 (12.5%)	0
Body weight [kg] ^#^	84.0 ± 19.3 (59.9–126.0)	99.6 ± 17.4 (75.0–126.0) ^1,2^	76.7 ± 9.8 (63.9–89.9) ^2^	64.7 ± 2.9 (59.9–67.0)
BMI [kg/m^2^] ^#^	30.3 ± 7.5 (21.4–47.6)	36.9 ± 6.8 (30.0–47.6) ^1,2^	27.0 ± 0.9 (25.7–28.6) ^2^	22.6 ± 1.4 (21.4–24.8)
Total fat tissue mass [kg] ^#^	31.9 ± 14.0 (14.0–60.5)	43.2 ± 11.7 (27.0–60.5) ^1,2^	21.7 ± 5.8 (14.0–34.6)	21.2 ± 3.6 (17.1–23.8)
Total fat tissue mass/body weight [%] ^#^	36.0 ± 8.7 (15.6–48.0)	42.7 ± 4.6 (36.0–48.0) ^1,2^	28.7 ± 7.3 (15.6–40.9)	32.7 ± 3.7 (28.5–35.7)
Visceral fat indicator ^#^	8.7 ± 4.1 (3–17)	11.0 ± 3.8 (6–17) ^1,2^	7.0 ± 3.5 (3–14)	5.3 ± 1.1 (4–6)
HOMA-IR	2.6 ± 2.5 (0.6–11.5)	3.9 ± 3.3 (0.6–11.4)	1.5 ± 1.0 (0.7–3.0)	1.9 ± 1.1 (0.7–3.5)
Fasting serum glucose [mg/dL] ^#^	85.0 ± 10.7 (65.0–108.1)	93.2 ± 8.7 (81.8–108.1) ^1,2^	77.2 ± 8.1 (65.0–88.3)	81.4 ± 5.6 (74.0–89.0)
HbA1c [%] ^#^	5.4 ± 0.4 (4.8–6.1)	5.6 ± 0.3 (5.1–6.1) ^1^	5.1 ± 0.3 (4.8–5.4)	5.3 ± 0.3 (5.0–5.7)
Serum HDL cholesterol [mg/dL] ^#^	63.2 ± 11.9 (43.6–87.0)	56.7 ± 9.5 (43.6–68.8) ^2^	63.1 ± 10.0 (53.0–79.0) ^2^	76.4 ± 9.1 (65.0–87.0)
Serum LDL cholesterol [mg/dL]	113.5 ± 31.3 (42.2–176.0)	104.0 ± 32.2 (42.2–150.9)	123.1 ± 19.1 (94.0–149.0)	117.0 ± 44.7 (69.0–176.0)
Serum triglycerides [mg/dL]	111.2 ± 60.0 (38.0–252.5)	130.8 ± 72,5 (38.0–252.5)	87.3 ± 23.6 (55.0–122.0)	110.2 ± 69.8 (49.0–190.0)
eGFR (CKD-EPI) [mL/min/1.73 m^2^]	82.6 ± 12.1 (65.2–115.1)	100.5 ± 20.0 (72.5–144.3)	92.8 ± 9.0 (79.7–105.4)	85.0 ± 5.4 (80.0–92.9)
Serum CRP [mg/L] ^#^	3.0 ± 4.2 (0.1–13.4)	5.8 ± 5.2 (0.6–13.4) ^1,2^	1.0 ± 0.9 (0.1–2.4)	0.7 ± 0.5 (0.1–1.3)

Means ± standard deviations (ranges); differences at *p* < 0.05 between the groups: * χ^2^ test; ^#^ Kruskal–Wallis Analysis of Variance with Mann–Whitney U test as post hoc: ^1^ *p* < 0.05 vs. overweight; ^2^ *p* < 0.05 vs. normal weight.

**Table 2:** Fractions of cultured cells positive for the Ad-MSC surface CD antigens in the study subgroups (obese: ≥30 kg/m^2^; overweight: 25–29.9 kg/m^2^; normal weight: <25 kg/m^2^; prediabetic: serum fasting glucose 100–125 mg/dL or HbA1c 5.7–6.4%; euglycemic: serum fasting glucose < 100 mg/dL and HbA1c < 5.7%).

	All Subjects *n* = 23	Obese *n* = 10	Overweight *n* = 8	Normal Weight *n* = 5	Obese	
					Prediabetic *n* = 6	Euglycemic *n* = 4
CD90	97.6 ± 3.0% (91.0–100%)	98.3 ± 3.0% (91.0–100%)	96.5 ± 3.1% (92.2–100%)	97.4 ± 2.8% (94.5–100%)	97.5 ± 3.6% (91.0–100%)	99.6 ± 0.7% (98.5–100%)
CD105	95.7 ± 4.0% (84.1–100%)	96.2 ± 4.8% (84.1–100%)	96.7 ± 1.3% (94.0–98.3%)	92.0 ± 4.3% (88.2–96.6%)	95.6 ± 5.8% (84.1–99.5%)	96.9 ± 3.3% (92.6–100%)
CD146	17.3 ± 9.4% (7.3–38.8%)	15.5 ± 10.7% (7.3–38.8%)	26.0 ± 3.5% (23.5–28.4%)	15.0 ± 2.3% (13.4–16.6%)	12.5 ± 3.7% (8.8–16.2%)	17.8 ± 14.3% (7.3–38.8%)

Means ± standard deviations (ranges); Kruskal–Wallis Analysis of Variance: *p* > 0.15 between the groups as per BMI; Mann–Whitney U test: *p* > 0.3 between the groups of obese subjects as per glycemic status.

**Table 3:** Ad-MSC expression of the four obesity-related microRNAs in the study subgroups.

	All Subjects *n* = 23	Obese *n* = 10	Overweight *n* = 8	Normal Weight *n* = 5	Obese	
					Prediabetic *n* = 6	Euglycemic *n* = 4
miR-21	2.13 ± 1.85 (0.29–7.59)	2.19 ± 2.35 (0.29–7.59)	1.55 ± 0.72 (0.41–2.49)	2.92 ± 2.00 (0.62–5.39)	1.23 ± 0.73 (0.29–2.18)	3.64 ± 3.32 (0.43–7.59)
miR-122	0.18 ± 0.13 × 10^−3^ (0.01–0.42 × 10^−3^)	0.19 ± 0.14 × 10^−3^ (0.01–0.42 × 10^−3^)	0.20 ± 0.15 × 10^−3^ (0.01–0.37 × 10^−3^)	0.12 ± 0.06 × 10^−3^ (0.05–0.21 × 10^−3^)	0.19 ± 0.17 × 10^−3^ (0.01–0.42 × 10^−3^)	0.19 ± 0.09 × 10^−3^ (0.07–0.28 × 10^−3^)
miR-155 *	6.29 ± 5.0 × 10^−3^ (1.11–20.12 × 10^−3^)	3.69 ± 2.67 × 10^−3^ (1.11–9.07 × 10^−3^) ^1,2^	7.07 ± 4.42 × 10^−3^ (3.04–15.4 × 10^−3^)	10.25 ± 7.05 × 10^−3^ (3.35–20.12 × 10^−3^)	3.08 ± 1.43 × 10^−3^ (1.35–4.98 × 10^−3^)	4.61 ± 4.01 × 10^−3^ (1.11–9.07 × 10^−3^)
miR-192	0.63 ± 0.24 × 10^−3^ (0.27–1.12 × 10^−3^)	0.63 ± 0.30 × 10^−3^ (0.29–1.12 × 10^−3^)	0.60 ± 0.26 × 10^−3^ (0.27–0.96 × 10^−3^)	0.65 ± 0.06 × 10^−3^ (0.55–0.71 × 10^−3^)	0.59 ± 0.23 × 10^−3^ (0.29–0.86 × 10^−3^)	0.71 ± 0.42 × 10^−3^ (0.29–1.12 × 10^−3^)

Means ± standard deviations (ranges); microRNA expression calculated as 2^−ΔCt^ values, expression related to SNORD61 control; * Kruskal–Wallis Analysis of Variance: *p* = 0.04 between the groups as per BMI; Mann–Whitney U test: ^1^ *p* = 0.06 vs. overweight; ^2^ *p* = 0.04 vs. normal weight; otherwise *p* > 0.1 between the groups.

**Table 4:** Coefficients of Spearman rank correlations (R_S_) or sex-adjusted partial correlations (r) of the studied Ad-MSC microRNA expressions with the clinical and metabolic parameters in the whole study group.

	Ad-MSC Expression of:			
	miR-21	miR-122	miR-155	miR-192
Age	R_S_ = −0.08 (r = −0.06)	R_S_ = −0.16 (r = −0.15)	R_S_ = −0.28 (r = −0.17)	R_S_ = −0.14 (r = −0.13)
Body weight	R_S_ = −0.12 (r = −0.16)	R_S_ = 0.10 (r = −0.01)	R_S_ = −0.49, *p* = 0.02 (r = −0.56, *p* < 0.01)	R_S_ = −0.18 (r = −0.16))
BMI	R_S_ = −0.21 (r = −0.16)	R_S_ = 0.08 (r = −0.01)	R_S_ = −0.65, *p* < 0.01 (r = −0.64, *p* < 0.01)	R_S_ = −0.19 (r = −0.20))
Total fat tissue mass	R_S_ = −0.11 (r = −0.11)	R_S_ = −0.10 (r = −0.04)	R_S_ = −0.49, *p* = 0.02 (r = −0.52, *p* = 0.02)	R_S_ = −0.28 (r = −0.24))
Total fat tissue mass/body weight	R_S_ = −0.15 (r = −0.10)	R_S_ = −0.12 (r = −0.16)	R_S_ = −0.42, *p* = 0.06 (r = −0.52, *p* = 0.02)	R_S_ = −0.22 (r = −0.35)
Visceral fat indicator	R_S_ = −0.09 (r = −0.12)	R_S_ = −0.04 (r = −0.07)	R_S_ = −0.55, *p* = 0.01 (r = −0.49, *p* = 0.03)	R_S_ = −0.36 (r = −0.27)
HOMA-IR	R_S_ = −0.18 (r = −0.04)	R_S_ = 0.19 (r = 0.26)	R_S_ = −0.32 (r = −0.23)	R_S_ = −0.20 (r = −0.09)
Fasting serum glucose	R_S_ = −0.08 (r = 0.04)	R_S_ = 0.16 (r = 0.27)	R_S_ = −0.21 (r = −0.18)	R_S_ = 0.12 (r = 0.27)
HbA1c	R_S_ = −0.03 (r = 0.04)	R_S_ = 0.18 (r = 0.20)	R_S_ = −0.12 (r = −0.09)	R_S_ = 0.05 (r = 0.13)
Serum HDL cholesterol	R_S_ = −0.11 (r = −0.09)	R_S_ = −0.21 (r = −0.06)	R_S_ = 0.39, *p* = 0.06 (r = 0.51, *p* = 0.02)	R_S_ = −0.11 (r = −0.10)
Serum LDL cholesterol	R_S_ = 0.16 (r = 0.30)	R_S_ = 0.02 (r = 0.17)	R_S_ = 0.08 (r = 0.09)	R_S_ = −0.01 (r = 0.05)
Serum triglycerides	R_S_ = 0.24 (r = 0.29)	R_S_ = 0.34 (r = 0.35)	R_S_ = −0.06 (r = −0.02)	R_S_ = 0.15 (r = 0.18)
eGFR (CKD-EPI)	R_S_ = −0.31 (r = −0.23)	R_S_ = −0.11 (r = −0.12)	R_S_ = −0.22 (r = −0.22)	R_S_ = −0.06 (r = −0.15)
Serum CRP	R_S_ = −0.23 (r = −0.29)	R_S_ = −0.02 (r = −0.14)	R_S_ = −0.61, *p* < 0.01 (r = −0.63, *p* < 0.01)	R_S_ = −0.11 (r = −0.07)

Spearman rank or partial correlations at *p* > 0.1 unless stated otherwise.

**Table 5:** Ad-MSC secretion of the four obesity-related protein regulators in the study subgroups.

	All Subjects *n* = 23	Obese *n* = 10	Overweight *n* = 8	Normal Weight *n* = 5	Obese	
					Prediabetic *n* = 6	Euglycemic *n* = 4
VEGF [ng/mL]	2.88 ± 0.89 (1.24–4.12)	2.55 ± 0.90 (1.24–3.93)	3.06 ± 1.02 (1.85–4.12)	3.24 ± 0.49 (2.75–4.07)	2.76 ± 0.61 (2.12–3.78)	2.22 ± 1.26 (1.24–3.93)
IGF-1 * [ng/mL]	1.60 ± 0.48 (0.90–2.43)	1.75 ± 0.35 ^1^ (1.19–2.11)	1.67 ± 0.60 (0.90–2.43)	1.18 ± 0.28 (0.90–1.55)	1.72 ± 0.35 (1.20–2.11)	1.80 ± 0.41 (1.19–2.07)
IL-6 [ng/mL]	2.09 ± 0.73 (0.34–4.21)	1.96 ± 0.19 (1.52–2.15)	1.95 ± 0.99 (0.34–4.21)	2.60 ± 0.86 (1.92–3.66)	1.93 ± 0.23 (1.52–2.15)	1.99 ± 0.10 (1.89–2.11)
IL-10 [pg/mL]	1.63 ± 0.64 (0.64–3.28)	1.56 ± 0.55 (0.88–2.52)	1.57 ± 0.58 (0.67–2.53)	1.86 ± 0.94 (0.64–3.28)	1.73 ± 0.65 (0.88–2.52)	1.32 ± 0.27 (1.05–1.66)

Means ± standard deviations (ranges); * Kruskal–Wallis Analysis of Variance: *p* = 0.09 between the groups as per BMI; Mann–Whitney U test: ^1^ *p* = 0.02 vs. normal weight; otherwise *p* > 0.15 between the groups.

**Table 6:** Coefficients of Spearman rank correlations (R_S_) or sex-adjusted partial correlations (r) of the studied Ad-MSC protein secretions with the clinical and metabolic parameters in the whole study group.

	Ad-MSC Secretion of:			
	VEGF	IGF-1	IL-6	IL-10
Age	R_S_ = −0.18 (r = −0.13)	R_S_ = 0.15 (r = 0.18)	R_S_ = 0.51, *p* = 0.01 (r = 0.41, *p* = 0.06)	R_S_ = −0.22 (r = −0.24)
Body weight	R_S_ = −0.35 (r = −0.36)	R_S_ = 0.43, *p* = 0.04 (r = 0.41, *p* = 0.06)	R_S_ = −0.06 (r = −0.14)	R_S_ = 0.01 (r = 0.09)
BMI	R_S_ = −0.29 (r = −0.30)	R_S_ = 0.48, *p* = 0.02 (r = 0.46, *p* = 0.03)	R_S_ = −0.06 (r = −0.15)	R_S_ = −0.16 (r = −0.04)
Total fat tissue mass	R_S_ = −0.38 (r = −0.37)	R_S_ = 0.07 (r = 0.42, *p* = 0.06)	R_S_ = 0.07 (r = −0.28)	R_S_ = 0.12 (r = 0.17)
Total fat tissue mass/body weight	R_S_ = −0.38 (r = −0.42, *p* = 0.07)	R_S_ = −0.02 (r = 0.34)	R_S_ = −0.09 (r = −0.31)	R_S_ = 0.11 (r = 0/0.05)
Visceral fat indicator	R_S_ = −0.30 (r = −0.27)	R_S_ = 0.50, *p* = 0.02 (r = 0.55, *p* = 0.01)	R_S_ = 0.18 (r = 0.03)	R_S_ = −0.19 (r = −0.03)
HOMA-IR	R_S_ = −0.24 (r = −0.26)	R_S_ = 0.18 (r = 0.26)	R_S_ = 0.11 (r = −0.08)	R_S_ = 0.15 (r = 0.13)
Fasting serum glucose	R_S_ = −0.52, *p* = 0.01 (r = −0.45, *p* = 0.04)	R_S_ = 0.21 (r = 0.45, *p* = 0.04)	R_S_ = 0.02 (r = −0.16)	R_S_ = 0.11 (r = 0.13)
HbA1c	R_S_ = −0.25 (r = −0.21)	R_S_ = 0.11 (r = 0.20)	R_S_ = 0.21 (r = 0.11)	R_S_ = 0.09 (r = 0.01)
Serum HDL cholesterol	R_S_ = −0.06 (r = 0.06)	R_S_ = −0.33 (r = −0.38, *p* = 0.08)	R_S_ = 0.23 (r = 0.33)	R_S_ = 0.25 (r = 0.29)
Serum LDL cholesterol	R_S_ = −0.05 (r = 0.01)	R_S_ = −0.35 (r = −0.29)	R_S_ = 0.19 (r = 0.33)	R_S_ = −0.09 (r = −0.22)
Serum triglycerides	R_S_ = −0.25 (r = −0.25)	R_S_ = 0.11 (r = 0.14)	R_S_ = 0.02 (r = −0.02)	R_S_ = 0.20 (r = 0.05)
eGFR (CKD-EPI)	R_S_ = −0.02 (r = 0.06)	R_S_ = 0.16 (r = 0.11)	R_S_ = −0.55, *p* < 0.01 (r = −0.45, *p* = 0.03)	R_S_ = −0.04 (r = −0.06)
Serum CRP	R_S_ = 0.06 (r = 0.03)	R_S_ = 0.43, *p* = 0.04 (r = 0.61, *p* < 0.01)	R_S_ = 0.08 (r = −0.17)	R_S_ = −0.04 (r = −0.28)

Spearman rank or partial correlations at *p* > 0.1 unless stated otherwise.

## Data Availability

The data presented in this study are available upon request from the corresponding author. The data are not publicly available to protect the confidentiality of the participating subjects.
